# A method to extract cytokines and matrix metalloproteinases from Schirmer strips and analyze using Luminex

**Published:** 2011-04-27

**Authors:** Karl R. VanDerMeid, Stephanie P. Su, Kathleen L. Krenzer, Keith W. Ward, Jin-Zhong Zhang

**Affiliations:** 1 Pharmaceutical R&D, Bausch + Lomb, Inc., Rochester, NY; 2 Research Clinic, Bausch + Lomb, Inc., Rochester, NY

## Abstract

**Purpose:**

The Schirmer’s test is commonly used in the clinic for the diagnosis of dry eye disease by measuring tear volume. This report describes a procedure which can be used to recover tears from the Schirmer strip for the measurement of multiple tear cytokines as well as matrix metalloproteinases (MMPs) by Luminex technology.

**Methods:**

Cytokine and MMP recovery was determined by using spiked Schirmer strips presoaked with known cytokines or MMPs prepared in PBS with 1% BSA. In a clinical study, tears were collected from 5 subjects using Schirmer strips. Strips were stored on ice immediately after removal from the subject and stored dry at −20 °C for 16–24 h. Cytokines were extracted from the Schirmer strip in 0.5 M NaCl with 0.5% Tween-20. Concentrations of cytokines and MMPs in collected tear samples were analyzed by Luminex using both a 10-cytokine and a 5-MMP kit.

**Results:**

The standard curves for the assay in both the kit assay buffer and extraction buffer were identical for 9 of the 10 cytokines and all 5 MMPs. In the clinical sample all the cytokines (interleukin 1α [IL-1α], IL-1β, IL-1ra, IL-4, IL-6, IL-8, IL-10, IL-13, monocyte chemotactic protein-1 [MCP-1], and tumor necrosis factor-α [TNF-α]) and 5 MMPs (MMP-1, MMP-2, MMP-7, MMP-9, and MMP-10) tested were detected in at least 50% of the 10 subject samples. Recoveries from extracted Schirmer strips were >60% for 8 of the 10 cytokines and all MMPs.

**Conclusions:**

Numerous cytokines and MMPs were detected in the tear samples collected using the Schirmer strip, including many that have been implicated in ocular surface disease. This procedure may be used to evaluate the cytokine and MMP content in tear samples in clinical studies, especially for the evaluation of dry eye therapeutics. Because the Schirmer test is routine in the assessment of dry eye, this method offers the opportunity to evaluate both the quantity and quality of the tears.

## Introduction

Tear samples are being used with increasing frequency to detect biomarkers of normal and diseased states of the ocular surface [1-3], including allergy [4], and dry eye [5,6]. While use of ELISA is common, the employment of multiplex bead [7,8], multi-array [9], and proteomic [10-12] technology has enhanced the use of collected tears by allowing for analysis of small sample volumes while increasing the number of detectable targets.

The most commonly reported method to collect tear samples for biomarker analysis is by capillary tube [13-15]. In addition, it has been reported that cellulose acetate absorbent filters [16], a Weck Cell Sponge [4], and an water eye wash [17] have been used to collect tear samples. However, Schirmer strips are routinely used to measure the tear volume for the clinical assessment of dry eye disease. It would be of added value if, rather than discarding the strips after the assessment, the tear components could be eluted from these strips and used for the measurement of known biomarkers, such as proinflammatory cytokines and other inflammatory mediators.

Cytokines, chemokines, and matrix metalloproteinases (MMPs) have been considered as potential biomarkers for ocular surface inflammation. Indeed, increased levels of pro-inflammatory cytokines such as interleukin 1 (IL-1), IL-6, and tumor necrosis factor-α (TNF-α), chemokines such as IL-8 and monocyte chemotactic protein-1 (MCP-1), and matrix metalloproteinase-9 (MMP-9) have been demonstrated to be associated with ocular surface diseases [1,4,18,19] including dry eye [20,21].

The Luminex multi-analyte profiling assay system is a technology based on the principle of flow cytometry. The system allows one to simultaneously measure numerous analytes in a single microplate well, using very small sample volumes [7] while achieving excellent correlations to individual ELISA for many cytokines [22]. This system has been successfully used to study the effect of anti-inflammatory agents on cytokine release profiles of cultured human ocular cells [23,24]. Recently, Luminex has also been used to measure the cytokine content in tear samples collected with capillary tubes [5]. The results from the current study demonstrate that numerous cytokines and MMPs can be detected by Luminex from the tear samples collected using a Schirmer strip.

## Methods

### Reagents

TearFlo™ Schirmer filter paper strips with an inked ruler were obtained from Contacare Ophthalmics & Diagnostics (Gujarat, India). Human multiplex-cytokine and MMP kits were from Millipore (Billerica, MA). All other reagents were purchased from standard commercial sources and were of the highest available purity.

### Standard curve of solution volume to Schirmer strip reading

To calculate millimeters of wetting with volume of tears collected, a standard curve was developed. Phosphate-buffered saline (PBS; 2.5, 5, 7.5, 10, 15, 20, 25, or 30 µl) was transferred to the rounded end of a Schirmer strip and the strip was placed in a 2-ml eppendorf tube lying flat on the bench surface. After 1 min the measurement as millimeter (mm) wetting was recorded. This measurement was repeated a total of 3 times for each volume tested, and a standard curve of volume to Schirmer strip reading was generated.

### Clinical subjects

The clinical study protocol was approved by the Southwest Independent Institutional Review Board and was conducted in accordance with 21 Code of Federal Regulations for Clinical Trials (CFR) Parts 812, 50, 54, and 56, applicable Bausch + Lomb Standard Operating Procedures, and the Declaration of Helsinki. All five healthy volunteers gave informed consent and they were assessed for eligibility. Inclusion criteria were as follows: be 18 years or older and have full legal capacity to volunteer, have no allergic conjunctivitis, not be using any topical ocular medications, no contact lens wear or ophthalmic drop use 8 h before their study visit, be willing and able to follow instructions, and have signed a statement of informed consent. Subject discontinuation criteria were: adverse effects, other ocular complications, subject non-compliance, subject request, or subject found to be ineligible during study participation. The age, gender, Ocular Surface Disease Index (OSDI), and Schirmer strip measurements for the five subjects are shown in Table 1.

**Table 1:** Clinical subject data.

**Subject number**	**Age**	**Gender**	OSDI*	**Severity based on OSDI**	**Schirmer OD**	**Schirmer OS**
1	54	F	37.5	1	10	15
2	41	F	25	1	6	7
3	26	M	0	0	28	32
4	53	F	20.8	1	6	14
5	25	F	0	0	35	35

*OSDI scale [34]: Normal (0) 0–20.7; Mild (1) 20.8–41.6; Moderate (2) 41.7–72.9; Severe (3) 73–100.

### Tear sample collection

The Investigator, with gloves, placed a Schirmer strip over the lid margin at the junction of the lateral and middle thirds of the lower eyelids and kept in place for 5 min while subjects closed their eyes without an anesthetic. The Schirmer strips were removed with gloves and tear volume in millimeters was recorded. Each Schirmer strip was placed into a sterile 2-ml centrifuge tube, stored on ice for 20 min to 1 h, and then stored at −20 °C until processed.

### Preparation of spiked cytokine and MMP Schirmer strips

To determine the feasibility and sensitivity of the kits for recovering cytokines and MMPs from the Schirmer strips, the low internal quality controls (QC-1) from each cytokine and MMP kit were prepared as described in the kit assay protocol. Twenty µl of each QC-1 was aliquotted to a Schirmer strip, allowed to flow for 1 min and the strip was transferred to a 2-ml eppendorf tube and frozen at −20 °C (24 h). For percent recovery, 20 µl of each QC was simultaneously aliquotted into a separate eppendorf tube and frozen at −20 °C (24 h). Each experiment for the strips and diluted frozen samples were prepared in triplicate.

### Extraction of cytokines and MMPs from Schirmer strips

Assay buffer (200 µl) of the analyte kit containing 1% BSA (BSA) in phosphate buffered saline (PBS) with sodium azide as a preservative or extraction buffer containing 0.5 M NaCl and 0.5% Tween-20 [25] was added to each 2-ml centrifuge tube and incubated for 3 h at ambient temperature on a rocker (VWR, West Chester, PA), and then stored on ice upon completion. The strip was transferred to a new 2-ml tube and residual liquid was removed by pinching the strip at the 25-mm mark in the sealed tube cap, and the sample was then centrifuged (Microfuge R, Beckman, Palo Alto, CA) at ~100× g for 10 s. This liquid was combined with stored extraction buffer. The Schirmer strips were discarded. Each 20-µl frozen sample was diluted in 180 µl assay or extraction buffer and treated to the same extraction regimen as described for the strips.

### Standard curve comparison

To ensure there was no interference in the assay from the extraction buffer, standard curves were prepared in parallel in the kit assay buffer and extraction buffer and were assessed on the same assay plate. Standard curves were generated using the kit internal standards *vs* maximum fluorescence intensity (MFI) and were graphically represented using Sigmaplot software (Systat, San Jose, CA).

### Statistical analysis of cytokine and MMP standard curves in both the assay buffer and extraction buffer

Comparison of each individual standard concentration points prepared in both the kit assay buffer and extraction buffer was performed using a one-way ANOVA-Tukey-Kramer test (JMP 7 software; SAS Institute, Cary, NC) and for each standard concentration point p<0.05 was pre-determined to be statistically significant.

### Luminex multiplex cytokine and MMP analysis

Cytokine and MMP content in both the spiked and tear extracted strips were analyzed using multiplex Luminex technology [7,26] and performed according to the manufacturer's instructions with all kit reagents, assay and wash buffers. Analysis was performed on cytokines and MMPs listed in Table 2 and Table 3. Briefly, 25 µl of each sample extract was incubated with antibody-coated capture beads overnight at 4 °C on a Titer Plate Shaker (Lab-Line Instrument, Park, IL). Washed beads were further incubated with biotin-labeled anti-human cytokine antibodies for 1 h at room temperature followed by incubation with streptavidin-phycoerythrin for 30 min and followed by an additional wash step. Beads were resuspended in Luminex flow buffer for 5 min and samples were analyzed using Luminex 200™ (Luminex, Austin, TX).

**Table 2:** Percent recovery of cytokines diluted in buffer or extracted from spiked Schirmer strips.

** **	**Recovery in assay buffer**		**Recovery in extraction buffer**	
**Cytokine**	**Diluted sample (Mean/SE)**	**Extracted SS sample (Mean/SE)**	**Diluted sample (Mean/SE)**	**Extracted SS sample (Mean/SE)**
IL-1α	77.8±4.7	66.9±4.0	84.6±5.6	79.8±4.2
IL-1β	81.5±6.2	59.0*±3.8	84.5±4.3	82.9±4.6
IL-1rα	86.0±4.5	67.7±3.5	74.5±5.3	68.5±3.5
IL-4	96.9±8.7	37.2±3.5	62.9±5.1	6.7±1.4
IL-6	82.5±3.5	68.2±2.7	78.2±3.3	67.1±3.3
IL-8	91.1±5.3	68.3*±4.3	89.3±4.9	76.8±4.2
IL-10	83.0±3.2	30.5*±2.8	BLD	BLD
IL-13	83.8±5.0	49.7*±2.6	85.2±8.2	29.4*±3.5
MCP-1	90.1±4.4	77.7±4.1	87.6±4.4	84.2±2.5
TNF-α	81.9±5.8	62.8±2.8	91.5±7.0	65.1*±4.3

*p<0.05 versus cytokine diluted in same assay buffer and calculated from same buffer standard curve. BLD: Below the limit of detection. n=10 for all data.

**Table 3:** Percent recovery of MMPs diluted in buffer or extracted from spiked Schirmer strips.

** **	**Recovery in assay buffer**		**Recovery in extraction buffer**	
**MMP**	**Diluted sample (Mean/SE)**	**Extracted SS sample (Mean/SE)**	**Diluted sample (Mean/SE)**	**Extracted SS sample (Mean/SE)**
MMP-1	92.1 ± 7.8	88.9 ± 4.0	91.1 ± 9.7	78.5 ± 7.5
MMP-2	94.3 ± 3.1	89.4 ± 5.1	86.8 ± 3.9	81.4 ± 5.0
MMP-7	93.6 ± 5.0	68.2* ± 5.1	86.7 ± 2.5	76.3 ± 17.3
MMP-9	96.7 ± 9.2	86.1 ± 8.5	100.2 ± 8.3	84.5 ± 6.8
MMP-10	87.0 ± 6.3	79.3 ± 2.7	94.4 ± 7.5	77.2 ± 3.4

*p<0.05 versus MMP from diluted sample with extracted and concentration calculated from equivalent buffers. n=10 for all data.

### Determination of percent recovery of cytokine from spiked Schirmer strips

For percent recovery analysis the median fluorescence intensity (MFI) was used to obtain the concentration of each cytokine in pg/ml based on the standard curve of each cytokine assayed by Luminex. Concentrations were estimated using the Statlia software (Brendan Technologies, Inc., Carlsbad, CA). Each sample was tested in duplicate and resulting concentrations were averaged. Samples with concentrations of cytokines outside the assay limits, as determined from the Statlia analysis, were excluded from further analysis. Cytokines recovered from both the diluted samples and spiked Schirmer strips were compared to the nominal concentrations of the QC-1 control. Percent recovery for each sample was determined by the equation: Percent recovery=(Mean of recovered sample analyte concentration/nominal analyte concentration)*100. The nominal analyte concentration was determined by performing the QC-1 control according to the kit instructions.

### Statistical analysis of spiked cytokine from Schirmer strips

Comparison of the recovered sample from spiked Schirmer strips to the sample diluted in buffer was performed using a one-way ANOVA-Tukey-Kramer test (JMP 7) and a p<0.05 was pre-determined to be statistically significant.

### Determination of pg/mlmeasured in tears obtained via Schirmer strips

The amount of each cytokine or MMP measured in the tears extracted from Schirmer strips was expressed as total recovery in picograms per milliliter (pg/ml) as follows: First, the calculated sample in pg/ml was multiplied by the total extraction sample volume (0.2 ml) to give total pg in the extracted sample. Final pg/ml based upon Schirmer volume was calculated by dividing total pg extracted by the calculated Schirmer strip volume (pg/µl) and multiplying by 1,000. Means and standard errors (SE) of the 10 samples were determined for each cytokine and MMP.

## Results

### Standard curve of solution volume to Schirmer strip reading

**Figure 1:**
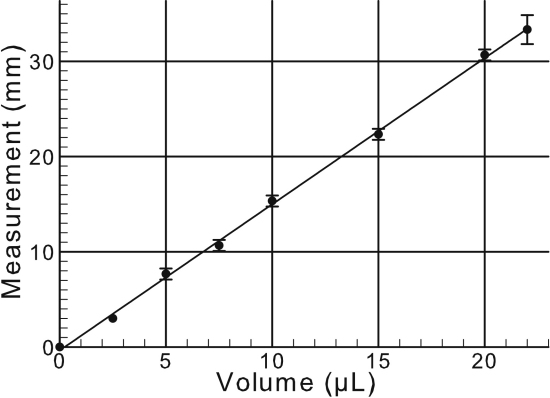
Graph of volume versus Schirmer strip measurement. Each data point represents the mean and standard deviation from 3 individual experiments. The line was determined by linear regression and the correlation coefficient was 0.999.

This experiment was designed to determine the relationship between the reading on the Schirmer strip as millimeter (mm) and actual volume (µl) of the PBS solution absorbed onto the strip over a period of one minute. Although a linear relationship was found, the tear volume measurement in millimeters did not equate to the same number for microliters (Figure 1). This standard curve was used to determine the actual volume of the tears on the clinical Schirmer strips.

### Luminex standard curves in standard assay and elution buffers

**Figure 2:**
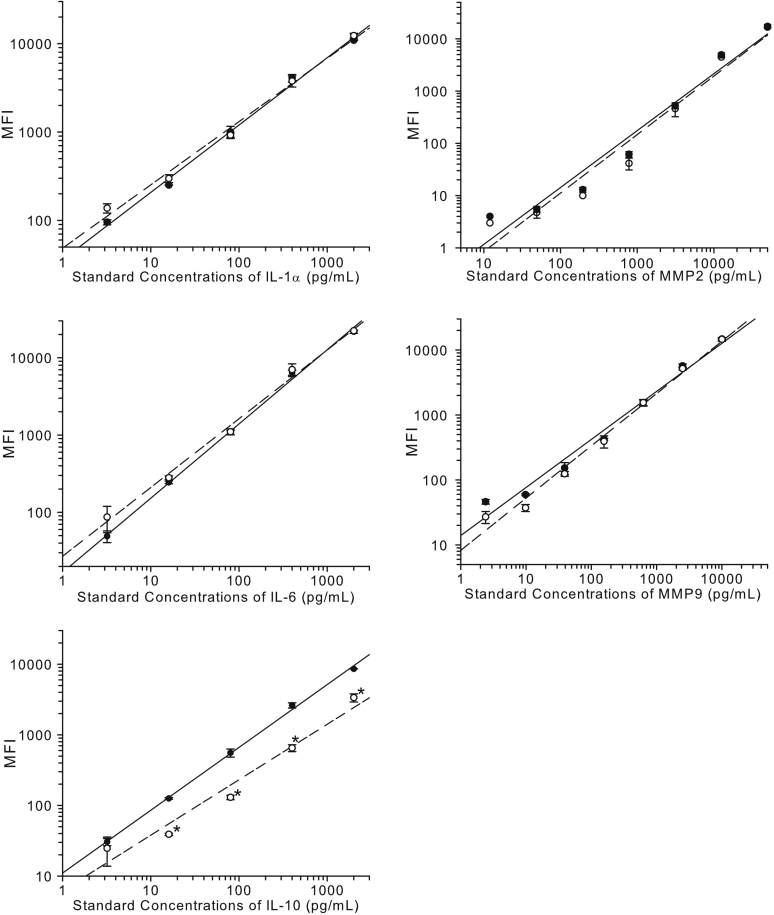
Extraction buffer containing high salt and detergent has no effect on Luminex standard curves. Luminex cytokine standard curve for preparations in kit assay buffer: closed circles with solid line or extraction buffer; open circles with dashed line. *p<0.05 versus equivalent concentration in assay buffer.

All the standard concentration points on curves generated in the extraction buffer for all cytokines and MMPs, except for IL-10, were statistically comparable to the concentration points generated in the kit assay buffer. For IL-10, all standard concentrations except for the lowest concentration were significantly lower in the extraction buffer. Graphs for IL-10, 2 major pro-inflammatory cytokines, IL-1α, and IL-6, and MMPs 2 and 9 are shown in Figure 2.

### Recovery of cytokines and MMPs from spiked Schirmer strips

For these experiments, and the clinical Schirmer strip extractions, identical 10 cytokine and 5 MMP kits were used. In these experiments the recovery for each 20-µl sample extracted from a Schirmer strip was compared to the recovery equal volume in solution. The percent recoveries represent the amount of diluted or extracted sample as compared to the theoretical recovery for each cytokine with each buffer. For the assays performed with kit assay buffer, the recoveries of IL-1β, IL-8, IL-10, and IL-13 were significantly lower in the extracted strips as compared to the diluted samples (Table 3). For the assays performed with the extraction buffer both IL-13 and TNF-α had significantly lower extracted strip recoveries, while IL-10 was below the limit of detection in this buffer and could not be analyzed. Of the extracted MMP strip samples, only MMP7, extracted in assay buffer had a significantly lower recovery.

### Recovery of cytokines and MMPs from clinical Schirmer strips

**Figure 3:**
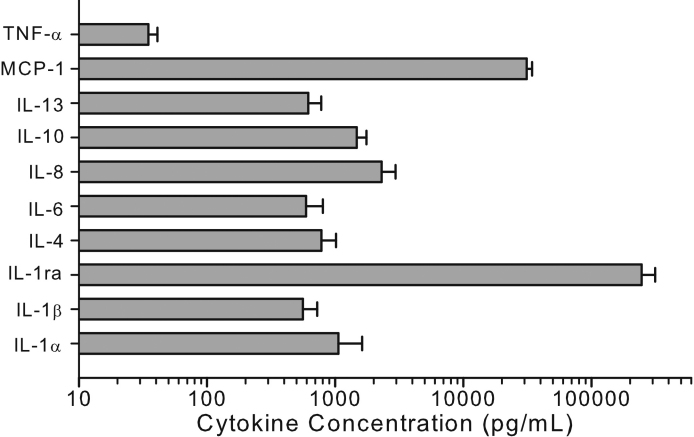
Concentrations of 10 cytokines extracted from clinical Schirmer strips. All data are expressed as mean±SEM.

**Figure 4:**
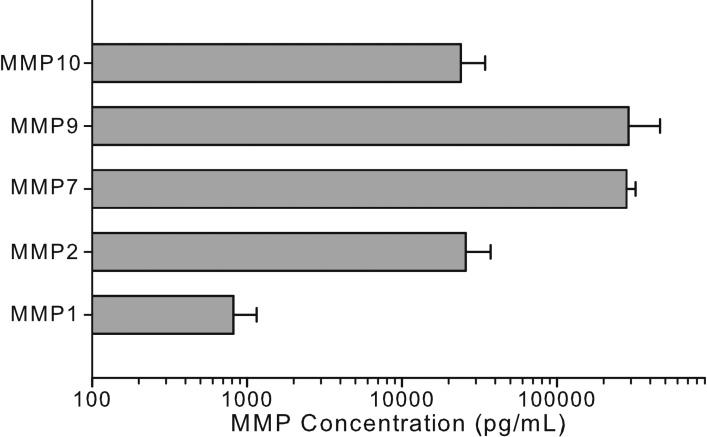
Concentrations of 5 MMPs extracted from clinical Schirmer strips. All data are expressed as mean±SEM.

Of the 10 samples tested in this study, all concentrations for the 10 cytokines tested were determined to be within the maximum reportable concentration except for 3 of the samples tested for IL-1ra (Figure 3). These 3 samples were re-tested at a 10-fold dilution in extraction buffer for IL-1ra only and the determined concentrations were found to be within the reportable range. Only 50% of samples for TNF-α were above the minimum detectable concentration for this assay, while all samples contained IL-1α, IL-1ra, IL-8 and MCP-1. Cytokines IL-6 and IL-13 were recovered in 80% of sample, IL-4 and IL-10 in 70% and IL-1β in 60%. Of the 5 MMPs tested in this study MMP-7, MMP-9, and MMP-10 were recovered in all the samples, while MMP-1 (70%) and MMP-2 (80%) were recovered in most of the samples (Figure 4). Only the samples within the reportable range of the assay were used for determination of calculated recoveries for these cytokines.

## Discussion

Researchers are using ever-expanding platforms of multiplex technology, which allow for the use of minimal sample volumes from tears to identify biomarkers associated with dry eye disease (DED) [6,9]. Schirmer’s test has been considered, rightly or wrongly [27,28], as a “gold standard” for the assessment of dry eye conditions, and is therefore a key test in both the clinical office setting and clinical trials for various treatment modalities. In addition to its traditional use of determining tear volume, this test also provides clinical samples which, until recently, have not been commonly assessed for comprehensive biologic alterations to the tear film in ocular surface disease. In part, this has been because of the relatively small amount of tears obtained as compared to the amount needed for biologic assays. However, with the advance of technologies which facilitate meaningful assessments in small-volume clinical samples, the potential for additional assessment from this standard test is very attractive. Recently, methods employed to collect tears from subjects include microcapillary tubes [8], polyvinyl acetate sponges [4], and acetate filters [16]. While each of these methods has its advantages, they would be an additional step in the clinical setting, costing additional time to both patient and clinical researcher. Furthermore, as both the Schirmer strip and the other techniques remove tears from the ocular surface, if both were performed; either procedure followed by the other would compromise the second test. Therefore, the use of Schirmer strips for additional biomarker analysis, including cytokines, chemokines, and MMPs, would be of immense benefit. Schimer strips have been used to successfully recover single analytes including eotaxins [25], cystatins [29], secretory IgA [30], and Vitamin C [31]. However, a comprehensive evaluation using Schirmer strips for the measurement of multiple cytokines as well as MMPs has not been previously described.

The Luminex multiplex platform is ideally suited for the detection of biomarkers from tear samples [6,8,28]. In the study presented here, Luminex kits were tested for capability of each kit to detect 10 cytokines and 5 MMPs recovered from spiked Schirmer strips. Initial experiments indicate that the prototype extraction buffer containing high salt and detergent does not interfere with the reproducible generation of standard curves for 14 of the 15 analytes tested. When the actual recovered sample from the spiked Schirmer strip was compared to the quantity recovered from the diluted control, both IL-10 and IL-13 were poorly recovered in both the assay and extraction buffers. It is unlikely that the observed poor recoveries of IL-10 and IL-13 were due to the buffer system since the two buffers tested in the current study were very different, one contains 0.5% Tween-20 and other one does not. Furthermore, cytokine kits from two other vendors were also tested and generated somewhat less efficient recoveries for most cytokines (data not shown). These findings suggest that a simple wetting of the strips may be sufficient to recover the cytokines and MMPs, and as our goal was to maximize the recovery of the most relevant pro-inflammatory cytokines, including IL-1, IL-6 and IL-8, the methods employed and the kit used in our studies were highly suitable for this purpose.

Finally, clinical Schirmer strip tear samples obtained from 5 subjects were tested for 10 cytokines of which 9 were recovered in >50% of the samples. However, while our clinical sample size was limited to 5 subjects, TNF-α was still below the limit of detection for half of these samples, suggesting either an inability of this cytokine to be detected using our methodology or that minimal quantities of this cytokine were present in our clinical test samples. Interestingly, IL-10, which was poorly recovered from spiked strips, was recovered in detectable quantities in most clinical samples. Additional work would be needed to further understand this phenomenon. In addition, all 5 MMPs were efficiently recovered from these samples, including MMP-9, which has been demonstrated to be involved in corneal barrier disruption in experimental dry eye models [32,33]. Future efforts will include the assessment of extraction buffer characteristics with regard to recovery and detection of cytokines, with emphasis on the primary pro- and anti-inflammatory cytokines and MMPs that may be affected in dry eye conditions. In addition, kits from multiple vendors may be used to develop our ability to detect the key cytokines in clinical samples.

Tear reflection is very common with Schirmer's test due to the strong irritation by the strip. By looking at Table 1, subject 5 and 3 might have tear reflection during the test, as their Schirmer's scores were abnormally high. With tear reflection, the cytokine and MMP can be easily diluted. Tear reflection was not considered in this study as the focus was more for the merits of the technical development on the cytokine recovery from the Schirmer strip than the actual concentrations of the cytokines recovered and the tear collection. However, this is a very important issue which should be considered when collecting tear samples and analyzing the cytokine concentration.

In summary, results from the current study demonstrated that numerous cytokines as well as several MMPs can be recovered from the Schirmer strip and quantitatively analyzed by Luminex technology. Because Schirmer’s test is routinely used for the assessment of dry eye and inflammation is recognized as a potential mechanism of dry eye or therapeutic target, this method offers the opportunity to evaluate both the quantity and quality of the tears.
